# Efficient generation and reversion of chromosomal translocations using CRISPR/Cas technology

**DOI:** 10.1186/s12864-016-3084-5

**Published:** 2016-09-17

**Authors:** Sergey Lekomtsev, Sofia Aligianni, Ana Lapao, Tilmann Bürckstümmer

**Affiliations:** 1 Horizon Discovery Ltd, Cambridge Research Park, Waterbeach, Cambridge, United Kingdom; 2 Horizon Genomics GmbH, Campus Vienna Biocenter, Vienna, Austria

**Keywords:** Gene fusions, Translocations, Chromosomal rearrangements, CRISPR, Cas9, Gene targeting, Philadelphia chromosome, BCR-ABL, CD74-ROS1, Cancer

## Abstract

**Background:**

Chromosomal translocations are a hallmark of cancer cells and give rise to fusion oncogenes. To gain insight into the mechanisms governing tumorigenesis, adequate model cell lines are required.

**Results:**

We employ the versatile CRISPR/Cas system to engineer cell lines in which chromosomal translocations are either generated *de novo* (CD74-ROS1) or existing translocations are reverted back to the original configuration (BCR-ABL1). To this end, we co-apply two guide RNAs to artificially generate two breakpoints and screen for spontaneous fusion events by PCR.

**Conclusions:**

The approach we use is efficient and delivers clones bearing translocationsin a predictable fashion. Detailed analysis suggests that the clones display no additional undesired alterations, implying that the approach is robust and precise.

**Electronic supplementary material:**

The online version of this article (doi:10.1186/s12864-016-3084-5) contains supplementary material, which is available to authorized users.

## Background

Chromosome abnormalities are a characteristic feature of cancer cells. Recurrent chromosome rearrangements, like translocations, are often found in various tumour types, and there is overwhelming evidence that they are key drivers of tumorigenesis [1]. Balanced chromosome translocations produce gene fusions, frequently leading to activation and overexpression of a gene due to the translocation. The nature of gene fusions strongly correlates with the tumour type, making them very attractive targets for cancer diagnostic or therapeutic intervention [2].

The first ever identified chromosome translocation in cancer was the Philadelphia chromosome originating from a balanced translocation between chromosomes 9 and 22 [1]. The Philadelphia chromosome is a characteristic feature of chronic myelogenous leukemia and was found in most of patients with this disease [3]. At the molecular level this translocation gives rise to the BCR-ABL1 fusion, thereby deregulating ABL1 tyrosine kinase activity. As a consequence, downstream signalling pathways are activated and malignant transformation of hematopoietic cells is induced [4]. The BCR-ABL1 gene fusion serves as a clinical biomarker for diagnostic and a therapeutic target for several drugs like imatinib, dasatinib, nilotinib and others [2].

Chromosome rearrangements involving ROS1 gene have been found in 1–2 % of patients with non-small cell lung cancers. Several fusion partners are known for ROS1, among which CD74 is the most common [5]. The CD74-ROS1 gene fusion is formed as result of a balanced translocation between chromosomes 5 and 6 in a way that preserves the tyrosine kinase domain of ROS1 [5]. As a result of this rearrangements, ROS1 kinase is constitutively active, rendering the CD74-ROS1 gene fusion a potent oncogenic driver [6].

Chromosomal translocations are triggered in vivo by the simultaneous occurrence of double strand breaks. To generate such translocations in defined cellular models, we used CRISPR/Cas technology [7, 8]. Cas9 is bacterial endonuclease derived from *Streptococcus pyogenes* that can be reprogrammed by short RNAs (so called guide RNAs) to induce a DNA double-strand break at any position that is followed by a protospacer adjacent motif (PAM) (NGG for *S. pyogenes* Cas9). Repair of the double-stand break can either occur by non-homologous end joining or by homology-directed repair, depending on whether a homology template is present or not. The CRISPR/Cas system has been used for genome engineering in a large number of species and for a wide variety of purposes (for review see [9]). The technology has also been used to engineer translocations in cells [10] or in mice [11, 12], but the overall efficiency by which clones were retrieved was not always apparent and the resulting cell lines were poorly characterized.

Here, we employed the haploid human cell line referred to as eHAP [13] as a model system to engineer or revert chromosomal translocations. We generated the CD74-ROS1 fusion *de novo* or reverted the BCR-ABL1 fusion that is naturally present in eHAP cells back to the wild-type state. Genotyping of hundreds of clones provides a measure for the overall efficiency of the process. Individual clones were characterized in great detail to assess the precision and robustness of the approach we devised.

## Results

**Fig. 1:**
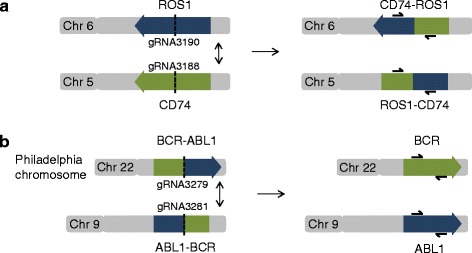
Strategy to induce translocations using CRISPR-Cas9. (**a**) The CD74-ROS1 rearrangement was generated by inducing a translocation between chromosomes 5 and 6. gRNA3190 and gRNA3188 were used to target wtCas9 nuclease to chromosome 6 and 5 respectively. Primers used for screening clones are shown. (**b**) BCR-ABL1 rearrangement was reverted by inducing translocation between chromosome 22 (Philadelphia chromosome) and 9. gRNA3279 and gRNA3281 were used to target wtCas9 nuclease to chromosome 22 and 9 respectively

To test the feasibility of engineering chromosomal translocations we attempted to generate the CD74-ROS1 gene fusion *de novo* (Fig. 1a). In parallel, we aimed to revert the existing BCR-ABL1 translocation back to wild-type state (Fig. 1b).

**Fig. 2:**
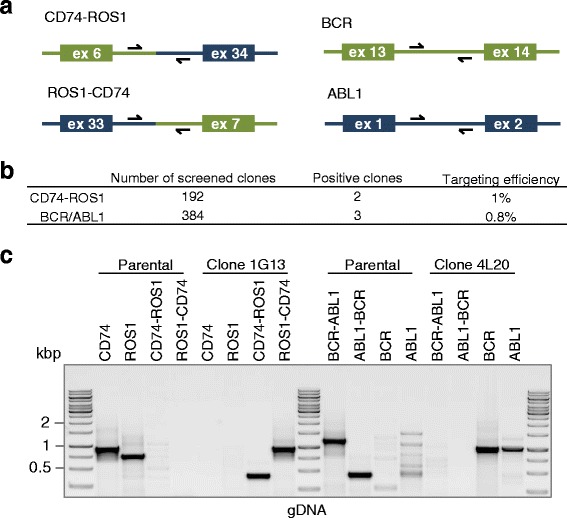
Chromosomal translocations are generated at high efficiency. (**a**) Schematic representation of DNA breakpoints and primers used for PCR amplification. (**b**) Overall efficiency of chromosomal translocations in eHAP cells. Clones were genotyped by PCR as shown in Additional file 2: Figure S2. (**c**) Agarose gel analysis of PCR amplified products. Individual clones bearing the CD74-ROS1 fusion (clone 1G13) or the BCR-ABL1 reversion (clone 4 L20) were characterized by PCR from genomic DNA. Primer sequences are specified in Additional file 3: Figure S3

In both cases, we used a similar approach. We applied Cas9 together with two guide RNAs, targeting the two breakpoints that we wished to create (Fig. 1, Additional file 1: Figure S1). Following enrichment of transfected cells using transient blasticidin selection, we isolated single cells by limiting dilution and genotyped clones for the presence/absence of the desired translocation. We screened a total number of 192 clones for CD74-ROS1 and 384 clones for BCR-ABL1 using PCR with primers spanning the newly generated junctions to identify clones harbouring translocation (Fig. 2a, Additional file 2: Figure S2 and Additional file 3: Figure S3). For CD74-ROS1, we recovered 2 clones (1.04 % targeting efficiency) that were positive for both the CD74-ROS1 and the reciprocal junction (Fig. 2b). For the reversion of BCR-ABL1, we identified 3 clones (0.78 % targeting efficiency) displaying the wild-type BCR and ABL1 junctions (Fig. 2b). We also show feasibility of this method in diploid cell line HEK293T (Fig. 1a and Additional file 4: Figure S4).

Mutant clones 1G13 (CD74-ROS1) and 4 L20 (BCR-ABL1 reversion) were selected for further analysis at the genomic DNA level. PCR analysis indicated that the mutant clones contained the desired translocation and that this translocation was absent in parental eHAP cells (Fig. 2c, Additional file 5: Figure S5). Conversely, the original sequences (CD74 and ROS1 wild-type or BCR-ABL1 and ABL1-BCR fusions) were only detectable in the parental cells, but not in the mutants. This indicates that the translocation occurred as expected and that each mutation/ translocation is present at a 100 % allele frequency in the resulting mutant eHAP cells.

**Fig. 3:**
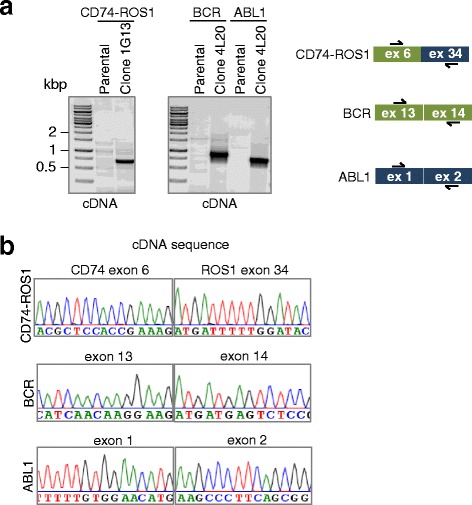
Gene fusions are detectable at the mRNA level. (**a**) Left panel: Total RNA was extracted from clones 1G13 (CD74-ROS1) or 4 L20 (BCR-ABL1 reversion) and analysed for the presence of CD74-ROS1 fusion or the presence of the BCR and ABL1 by non-quantitative RT-PCR. Right panel: schematic representation of generated cDNAs and primers used for PCR amplification. (**b**) Sanger sequencing of rearranged cDNA in clones 1G13 (CD74-ROS1) or 4 L20 (BCR-ABL1 reversion)

We also assessed whether the CD74-ROS1 translocation and BCR and ABL1 were detectable at the mRNA level. Clearly, the fusion of CD74 exon 6 to ROS1 exon 34 was detected by RT-PCR in the clone 1G13. Likewise, both BCR and ABL1 expression were restored in the clone 4 L20 (Fig. 3a). Sanger sequencing of the RT-PCR products indicated that exons were fused as predicted without additional insertions or deletion (Fig. 3b). Given the fact that the DNA double strand breaks/translocations occurred in intronic sequences, this was expected.

**Fig. 4:**
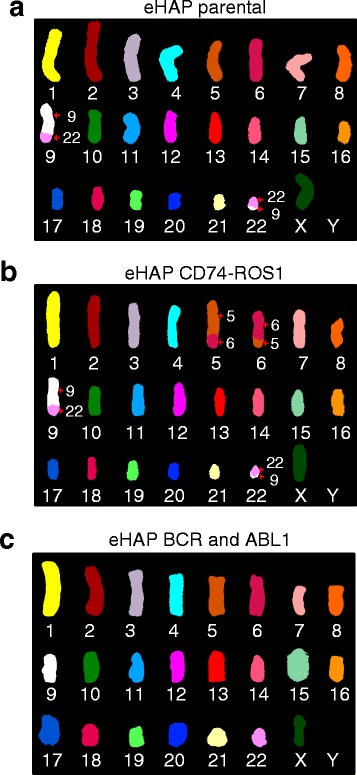
Clonal cell lines bear no additional genomic alterations. eHAP parental cells (**a**) or clones bearing the CD74-ROS1 fusion (clone 1G13; **b**) or the BCR-ABL1 reversion (clone 4 L20; **c**) were analysed by spectral karyotyping

We used spectral karyotyping [14] and G-band karyotyping to visualize the engineered translocations at the chromosomal level and to also assess whether additional undesired gross rearrangements had occurred. Spectral karyotyping and G-band staining revealed that the parental cell line eHAP contained intact chromosomes 5 and 6 and the reciprocal translocation between chromosomes 9 and 22 that give rise to the Philadelphia chromosome (Fig. 4a). Clone 1G13, which contains the CD74-ROS1 fusion, acquired a balanced translocation between the long arms of chromosome 5 and 6 (Fig. 4b, Additional file 6: Figure S6a). Clone 4 L20, in which we reverted the BCR-ABL1 fusion, displayed intact chromosomes 9 and 22, confirming the reversion of BCR-ABL1 translocation at the chromosomal level (Fig. 4c, Additional file 6: Figure S6b). Importantly, spectral karyotyping showed that engineered clones did not acquire additional chromosomal rearrangements due to potential CRISPR/Cas9 off-target activity. Taken together, these results show that CRIPSR/Cas9 system can be used to efficiently generate gross chromosomal rearrangements in human cells.

## Discussion

Chromosomal translocations underlie a variety of malignancies, yet definitive experimental models are missing. Here, we use the haploid human cell line eHAP to generate or revert such translocations. The method we use is efficient and works with surgical precision both in haploid and diploid backgrounds. By combining two guide RNAs, we introduce two simultaneous double strand breaks that get spontaneously joined by the endogenous DNA damage repair machinery. We speculate that Cas9 may serve as a bridging function as it is needed to generate both breaks. Consequently, the spontaneous fusion rate may be enhanced by using a Cas9 that can be dimerized, e.g. using the FK506 binding protein 12 (FKBP) and FKBP rapamycin binding (FRB) domain system [15]. Alternatively, the two ends may be bridged using an oligonucleotide that can hybridize to both ends of the junction that one wishes to establish [16].

While the high efficiency we observed is desired when engineering chromosomal translocations, it may be undesired in other instances, e.g. when engineering double knockouts. We suspect that at least in some instances, the simultaneous delivery of two guide RNAs will cause translocations and other genomic rearrangements as unwanted side effects. While these can easily be assessed in clonal cell lines that proliferate almost indefinitely, they may be more difficult to detect in scenarios where cell populations are more heterogeneous.

We used the haploid human cell line eHAP for the experiments presented here. In our view, this choice offers three major advantages, all of which are based on the fact that the cell carries a single genome copy: (i) Genotyping is a lot more straightforward than in diploid cells, (ii) Reversion back to wild-type configuration is hampered because a suitable homology template is missing and (iii) Cell lines derived contain 100 % allele burden with regard to the chromosomal translocation. However, we believe that this approach would be equally feasible in diploid cells. To exemplify this, we transduced diploid 293 T cells with two gRNAs for CD74 and ROS1 and detected both the CD74-ROS1 fusion and the reciprocal ROS1-CD74 fusion by PCR (Additional file 4: Figure S4). Whereas most people so far use the CRISPR technology for research applications, the technology also offers the potential to develop new therapies. As many translocations represent driver events of tumorigenesis (as opposed to passenger events), it is conceivable that CRISPR-mediated reversion of an oncogenic fusion event may become a therapeutic strategy in the distant future.

## Conclusion

The diagnosis of cancer is increasingly based on the characterization of the underlying genomic events. For these diagnostic procedures, robust reference materials that allow the standardization across laboratories are largely missing. We envisage that cell lines generated here and in future will be useful as a source of genomic DNA or mRNA that can be utilized for this purpose, alongside with DNA from an isogenic wild-type cell line. Implementation of such reference standards is likely to increase the quality of diagnostic output and will thus represent an important advancement in the era of personalized medicine.

## Methods

### Genome editing

We used an expression plasmid in which Cas9 from Streptococcus pyogenes was expressed from a CMV promoter. Guide RNA sequences are shown in Additional file 1: Figure S1 (gRNA3188 GGTCAAAGGGCCACCCTGCC, gRNA3190 GTTAAATTTAGTTGAAGCAC, gRNA3279 TGTAGGTAGTTGTACCAATA and gRNA3281 GACCTGTCTTTTAGACAGGC). Genome editing in eHAP cells was performed as described previously [13]. In brief, we transfected eHAP cells in 6 well plates using Turbofectin (Origene). Cells were transfected with 1 μg of Cas9 expression vector and 0.8 μg of guide RNA expression vector. To enrich for transfected cells, we cotransfected 0.2 μg of a plasmid encoding a blasticidin resistance gene and subjected cells to transient selection with 20 μg/ml blasticidin for 24 h. Transfected cells were expanded for limiting dilution after 2–3 days after blasticidin selection.

### Isolation of single-cell clones

Single eHAP clones were obtained by limiting dilution. Cells were trypsinized and serially diluted to a concentration of 15 cells per mL. Fifty microliters of this suspension were seeded in each well of a 384-well plate. Plates were imaged on day 0 and 7 after dilution using Cell Metric™ CLD imaging system (Solentim) to guaranty cell line clonality. Monoclonal cell lines were expanded using Microlab STAR Line robotic system (Hamilton Company).

### Genomic DNA isolation and PCR

For the primary PCR screen, genomic DNA was prepared using DirectPCR Lysis reagent (PeqLab) containing Proteinase K (Sigma). Genomic DNA was isolated from selected clones using the Gentra Puregene Cell Kit (Qiagen) according to manufacturer’s instructions. PCR was performed using GoTaq Polymerase (Promega) according to manufacturer’s instructions. PCR products were analysed using 1 % TAE agarose.

### RT-PCR

Total RNA was purified using RNAeasy Plus Mini Kit (Qiagen) and cDNA was synthesized using SuperScript® III Reverse Transcriptase (Thermo Fisher Scientific) according to manufacturer’s instructions.

### Spectral karyotyping

Spectral karyotyping was performed and analyzed by WiCell Cytogenetics. In brief, metaphase chromosome spreads were prepared on slides according to standard cytogenetic procedures. These slide preparations were then hybridized according to the DNA spectral karyotyping hybridization and detection protocol provided by Applied Spectral Imaging (ASI) using the supplied SKY probes and detection system. Images were captured and analysed using the HiSKYV spectrum imaging system from ASI.

## Additional files

Additional file 1: Figure S1. DNA sequences of targeted genomic loci and position of used gRNAs. (A) Sequences of CD74 and ROS1 targeted introns. (B) Genomic sequences of BCR-ABL1 and ABL1-BCR rearrangements. (PPTX 202 kb) Additional file 2: Figure S2. Original PCR screens for CD74-ROS1 translocation and BCR-ABL1 reversion (only results for 96 clones are shown for each rearrangement). (A) PCR screen on gDNA for clones harbouring CD74-ROS1 and ROS1-CD74 rearrangements. Asterisk indicates positive clone. (B) PCR screen on gDNA for clones harbouring repaired BCR and ABL1 genes. (PPTX 1609 kb) Additional file 3: Figure S3. Primers used for gDNA and cDNA amplification. (PPTX 38 kb) Additional file 4: Figure S4. Generation of CD74-ROS1 fusion in HEK293T cell line. We applied Cas9 together with two guide RNAs, targeting the two breakpoints that we aimed to create. We genotyped pooled cells for the presence of the CD74-ROS1 translocation with primers (Fig 1a) spanning the newly generated junctions. (PPTX 96 kb) Additional file 5: Figure S5. Sequences of gDNA junctions. (A) gDNA sequence of CD74-ROS1 fusion junction in 1G13 clone. Red rectangle indicates deletion. (B) gDNA sequence of ROS1-CD74 fusion junction in 1G13 clone. Red rectangle indicates insertion. (C) Repaired genomic sequence of BCR gene in 4 L20 clone. (D) Repaired genomic sequences of ABL1 gene in 4 L20 clone. (PPTX 314 kb) Additional file 6: Figure S6. G-band staining. (A) Clone 1G13 containing CD74-ROS1 fusion. (B) Clone 4 L20 containing intact BCR and ABL1 genes. (PPTX 271 kb)

## Abbreviations

cDNA: Coding DNA
CRISPR: Clustered regularly interspaced short palindromic repeat
FKBP: FK506 binding protein 12
FRB: FKBP rapamycin binding
gDNA: Genomic DNA
mRNA: Messenger RNA
PAM: Protospacer adjacent motif
RT-PCR: Reverse transcription polymerase chain reaction
sgRNA: Single guide RNA.
