# Supplementary figures and images for: Efficient generation and reversion of chromosomal translocations using CRISPR/Cas technology
Source: BMC Genomics. 2016 Sep 17;17:739. doi: 10.1186/s12864-016-3084-5 (PMC5027121; doi:10.1186/s12864-016-3084-5)

## Slide 1
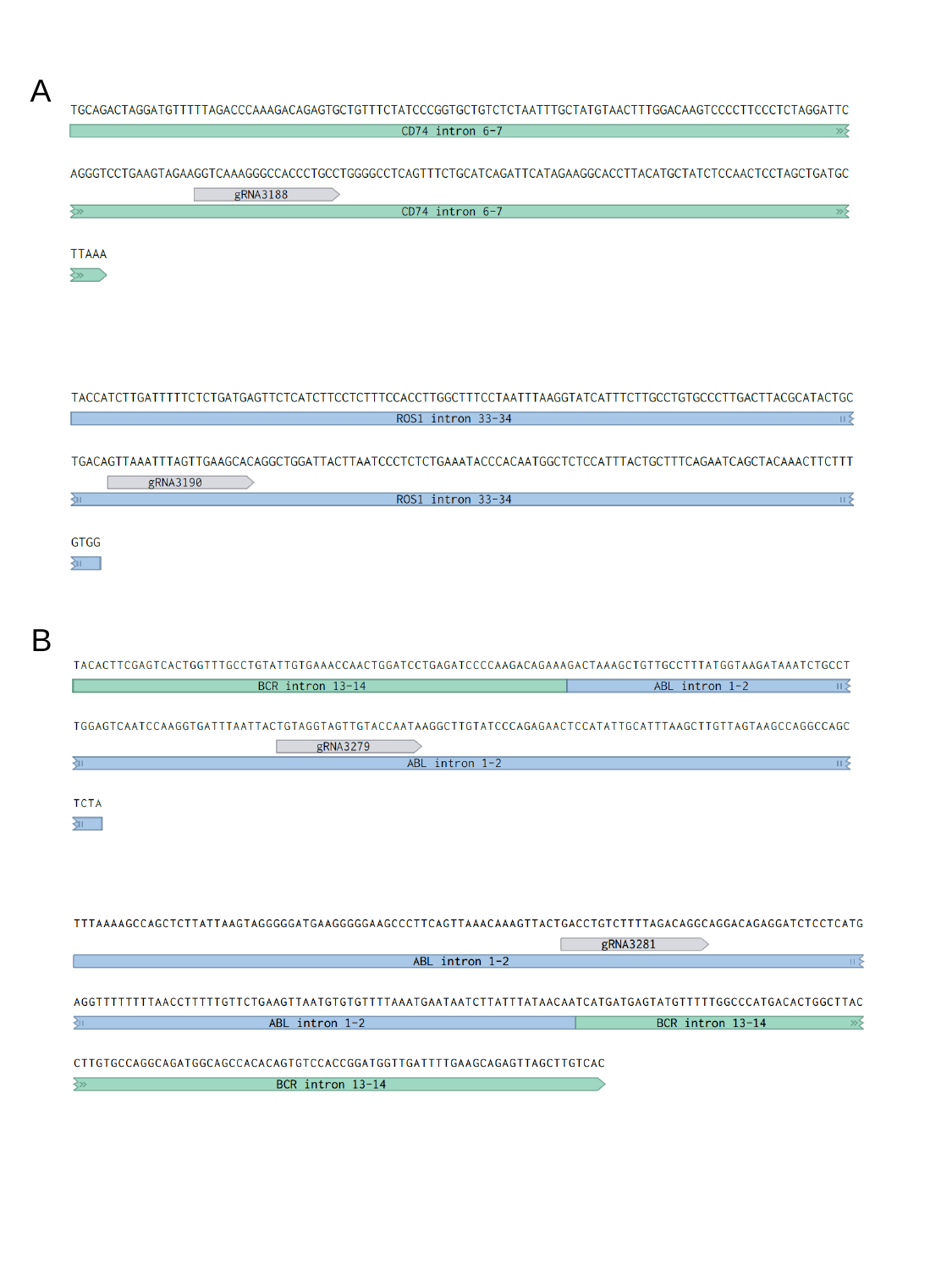

A
B

Supplement: Additional file 1: Figure S1. — DNA sequences of targeted genomic loci and position of used gRNAs. (A) Sequences of CD74 and ROS1 targeted introns. (B) Genomic sequences of BCR-ABL1 and ABL1-BCR rearrangements. (PPTX 202 kb) [file 12864_2016_3084_MOESM1_ESM.pptx]

## Slide 1
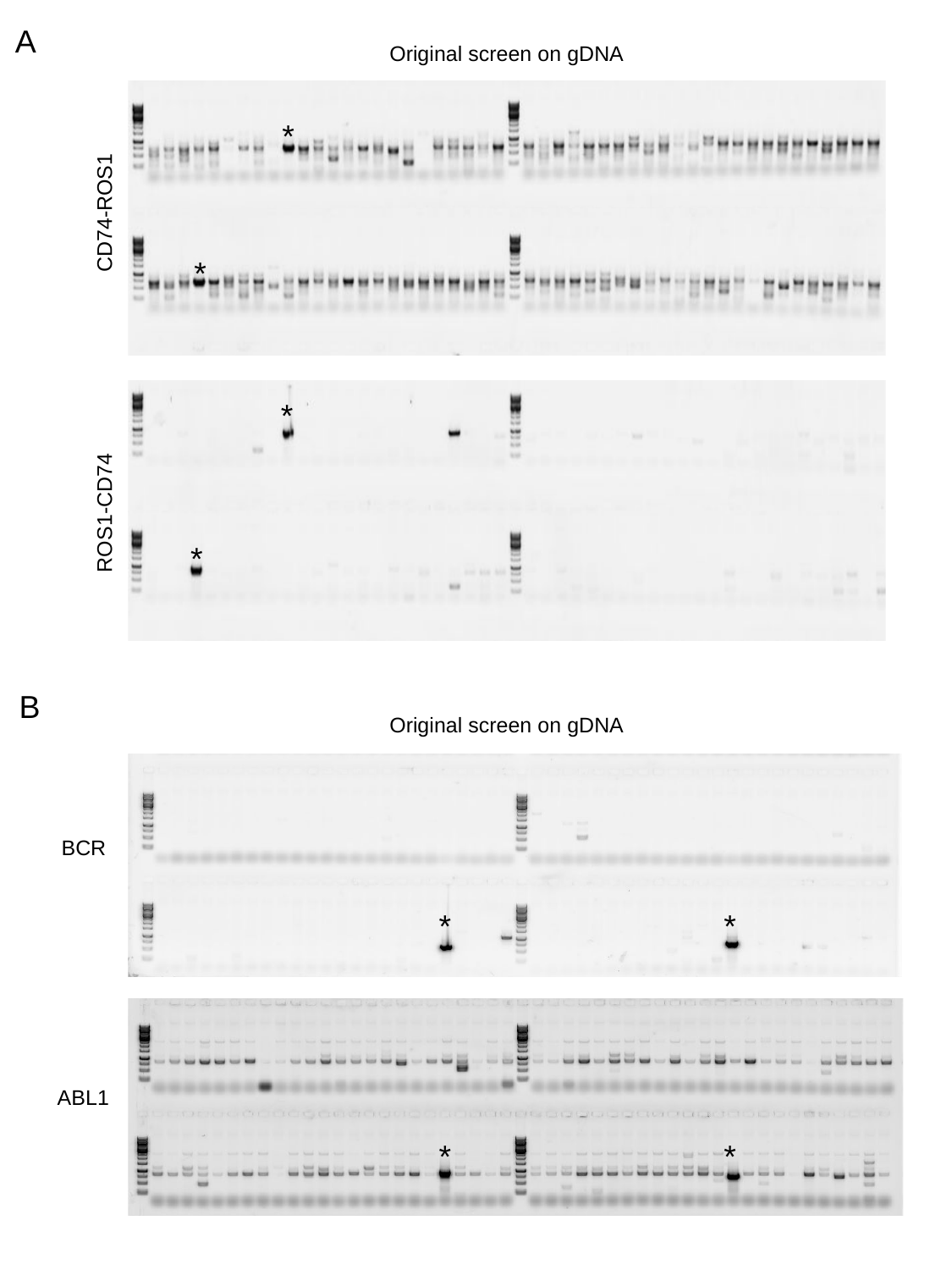

A
Original screen on gDNA
*
CD74-ROS1
*
*
ROS1-CD74
*
B
Original screen on gDNA
BCR
*
*
ABL1
*
*

Supplement: Additional file 2: Figure S2. — Original PCR screens for CD74-ROS1 translocation and BCR-ABL1 reversion (only results for 96 clones are shown for each rearrangement). (A) PCR screen on gDNA for clones harbouring CD74-ROS1 and ROS1-CD74 rearrangements. Asterisk indicates positive clone. (B) PCR screen on gDNA for clones harbouring repaired BCR and ABL1 genes. (PPTX 1609 kb) [file 12864_2016_3084_MOESM2_ESM.pptx]

## Slide 1
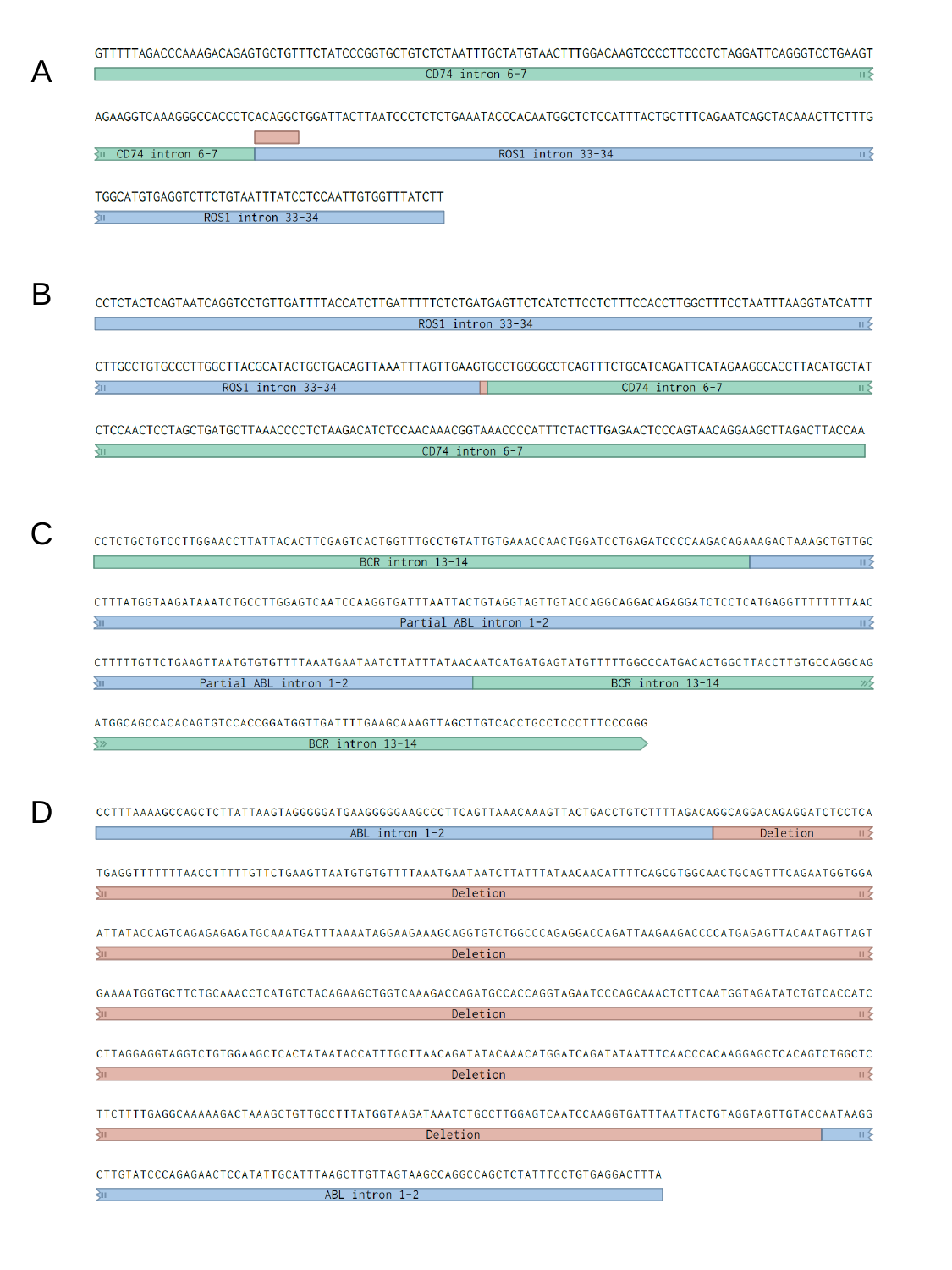

A
B
C
D

Supplement: Additional file 5: Figure S5. — Sequences of gDNA junctions. (A) gDNA sequence of CD74-ROS1 fusion junction in 1G13 clone. Red rectangle indicates deletion. (B) gDNA sequence of ROS1-CD74 fusion junction in 1G13 clone. Red rectangle indicates insertion. (C) Repaired genomic sequence of BCR gene in 4 L20 clone. (D) Repaired genomic sequences of ABL1 gene in 4 L20 clone. (PPTX 314 kb) [file 12864_2016_3084_MOESM5_ESM.pptx]

## Slide 1
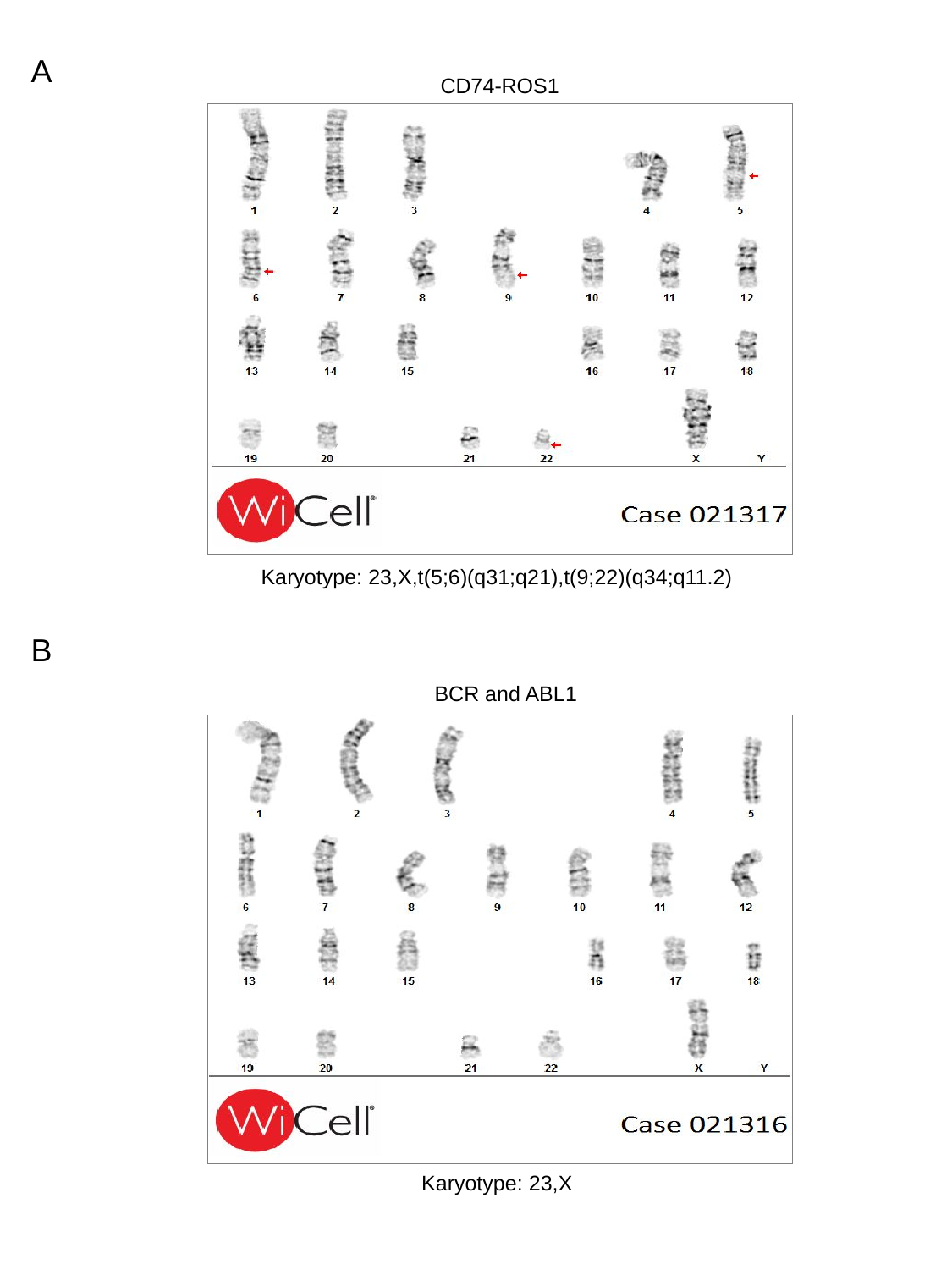

A
CD74-ROS1
Karyotype: 23,X,t(5;6)(q31;q21),t(9;22)(q34;q11.2)
B
BCR and ABL1
Karyotype: 23,X

Supplement: Additional file 6: Figure S6. — G-band staining. (A) Clone 1G13 containing CD74-ROS1 fusion. (B) Clone 4 L20 containing intact BCR and ABL1 genes. (PPTX 271 kb) [file 12864_2016_3084_MOESM6_ESM.pptx]
