# Supplementary material for: Efficient generation and reversion of chromosomal translocations using CRISPR/Cas technology
Source: BMC Genomics. 2016 Sep 17;17:739. doi: 10.1186/s12864-016-3084-5 (PMC5027121; doi:10.1186/s12864-016-3084-5)
Supplement: Additional file 3: Figure S3. — Primers used for gDNA and cDNA amplification. (PPTX 38 kb) [file 12864_2016_3084_MOESM3_ESM.pptx]

## Slide 1
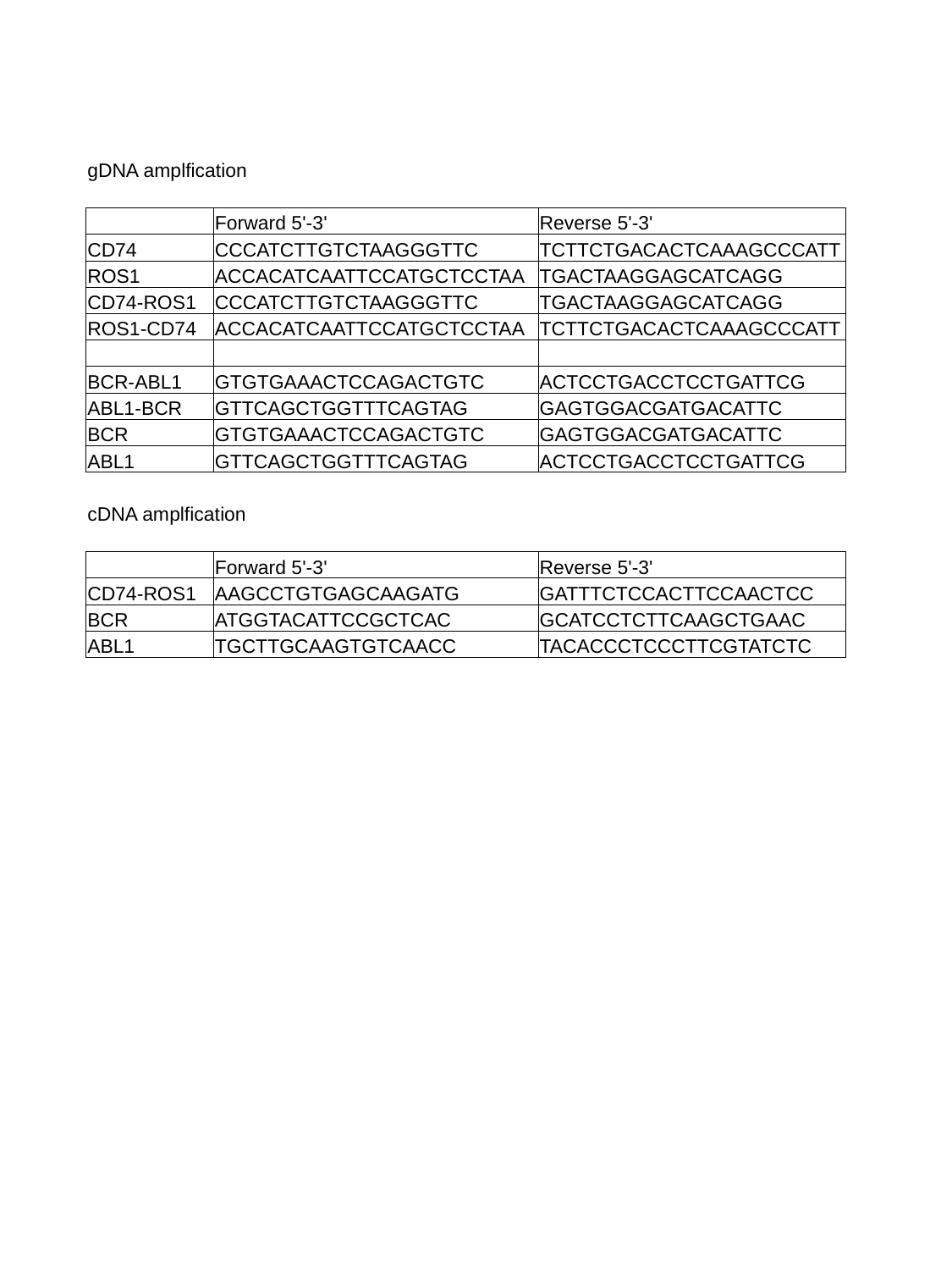

| gDNA amplfication | | |
| --- | --- | --- |
| | | |
| | Forward 5'-3' | Reverse 5'-3' |
| CD74 | CCCATCTTGTCTAAGGGTTC | TCTTCTGACACTCAAAGCCCATT |
| ROS1 | ACCACATCAATTCCATGCTCCTAA | TGACTAAGGAGCATCAGG |
| CD74-ROS1 | CCCATCTTGTCTAAGGGTTC | TGACTAAGGAGCATCAGG |
| ROS1-CD74 | ACCACATCAATTCCATGCTCCTAA | TCTTCTGACACTCAAAGCCCATT |
| | | |
| BCR-ABL1 | GTGTGAAACTCCAGACTGTC | ACTCCTGACCTCCTGATTCG |
| ABL1-BCR | GTTCAGCTGGTTTCAGTAG | GAGTGGACGATGACATTC |
| BCR | GTGTGAAACTCCAGACTGTC | GAGTGGACGATGACATTC |
| ABL1 | GTTCAGCTGGTTTCAGTAG | ACTCCTGACCTCCTGATTCG |
| | | |
| cDNA amplfication | | |
| | | |
| | Forward 5'-3' | Reverse 5'-3' |
| CD74-ROS1 | AAGCCTGTGAGCAAGATG | GATTTCTCCACTTCCAACTCC |
| BCR | ATGGTACATTCCGCTCAC | GCATCCTCTTCAAGCTGAAC |
| ABL1 | TGCTTGCAAGTGTCAACC | TACACCCTCCCTTCGTATCTC |
