# Supplementary material for: Efficient generation and reversion of chromosomal translocations using CRISPR/Cas technology
Source: BMC Genomics. 2016 Sep 17;17:739. doi: 10.1186/s12864-016-3084-5 (PMC5027121; doi:10.1186/s12864-016-3084-5)
Supplement: Additional file 4: Figure S4. — Generation of CD74-ROS1 fusion in HEK293T cell line. We applied Cas9 together with two guide RNAs, targeting the two breakpoints that we aimed to create. We genotyped pooled cells for the presence of the CD74-ROS1 translocation with primers (Fig 1a) spanning the newly generated junctions. (PPTX 96 kb) [file 12864_2016_3084_MOESM4_ESM.pptx]

## Slide 1
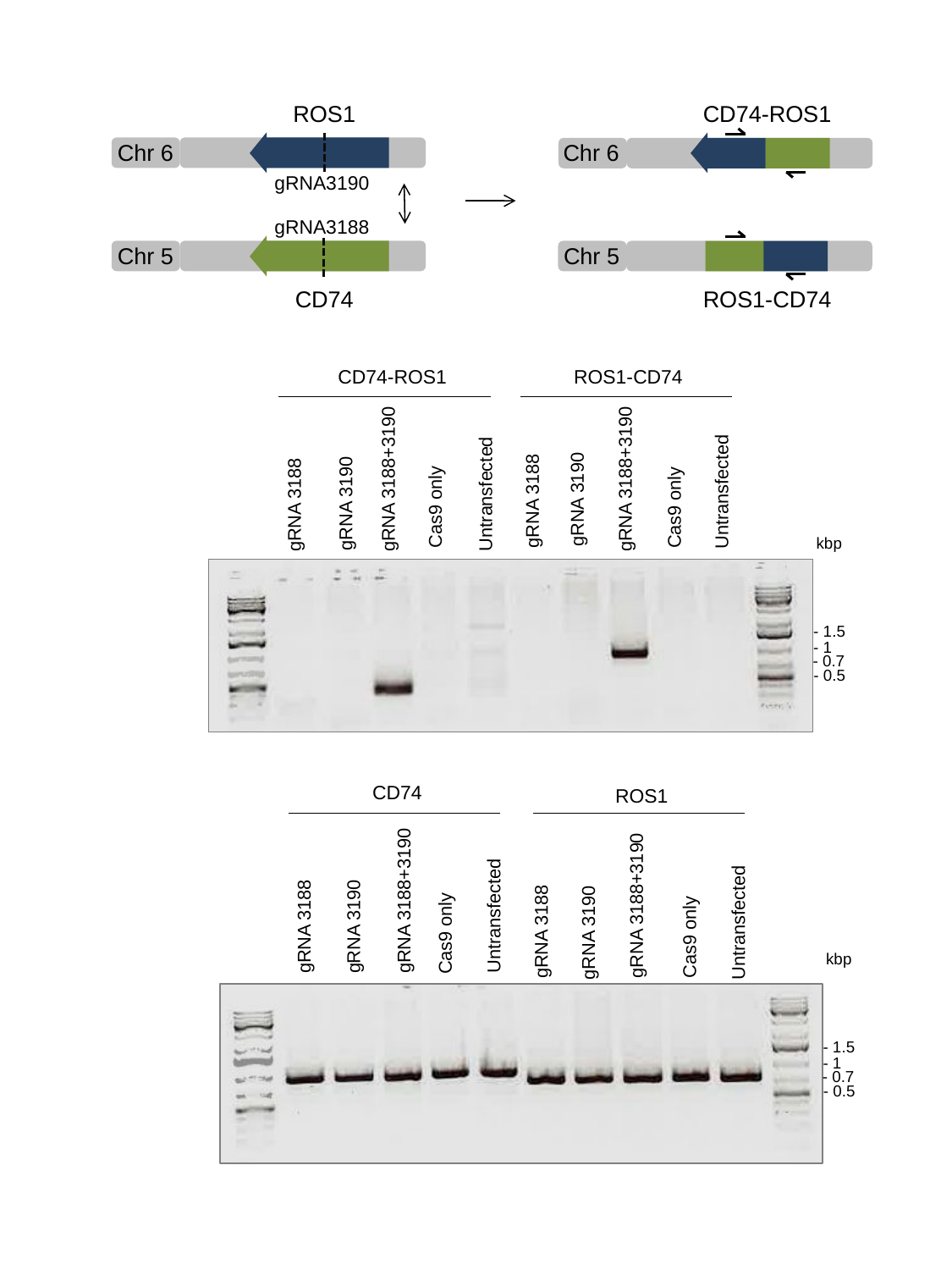

ROS1
CD74-ROS1
Chr 6
Chr 6
gRNA3190
gRNA3188
Chr 5
Chr 5
CD74
ROS1-CD74
CD74-ROS1
ROS1-CD74
gRNA 3188+3190
gRNA 3188+3190
Untransfected
Untransfected
gRNA 3190
gRNA 3190
gRNA 3188
gRNA 3188
Cas9 only
Cas9 only
kbp
- 1.5
- 1
- 0.7
- 0.5
CD74
ROS1
gRNA 3188+3190
gRNA 3188+3190
Untransfected
Untransfected
gRNA 3190
gRNA 3190
gRNA 3188
gRNA 3188
Cas9 only
Cas9 only
kbp
- 1.5
- 1
- 0.7
- 0.5
